# Heat Waves Occurrence and Outdoor Workers’ Self-assessment of Heat Stress in Slovenia and Greece

**DOI:** 10.3390/ijerph16040597

**Published:** 2019-02-19

**Authors:** Tjaša Pogačar, Zala Žnidaršič, Lučka Kajfež Bogataj, Andreas D. Flouris, Konstantina Poulianiti, Zalika Črepinšek

**Affiliations:** 1 Centre of Agrometeorology, Department of Agronomy, Biotechnical Faculty, University of Ljubljana, Jamnikarjeva 101, 1000 Ljubljana, Slovenia; lucka.kajfez.bogataj@bf.uni-lj.si (L.K.B.); zalika.crepinsek@bf.uni-lj.si (Z.Č.); 2 Faculty of Mathematics and Physics, University of Ljubljana, Jadranska 19, 1000 Ljubljana, Slovenia; zala.znidarsic@gmail.com; 3 FAME Laboratory, Department of Exercise Science, University of Thessaly, Karies, 42100 Trikala, Greece; andreasflouris@gmail.com (A.D.F.); poulianitikon@gmail.com (K.P.); 4 Human and Environmental Physiological Research Unit, Faculty of Health Sciences, University of Ottawa, Ottawa, ON K1N 6N5, Canada

**Keywords:** heat wave, outdoor workers, occupational health, heat stress, climate change, productivity

## Abstract

Changing patterns of heat waves are part of the global warming effect and the importance of changes is reinforced by their negative impact on society. Firstly, heat waves were analyzed in Brnik (Slovenia) and Larisa (Greece) in the period 1981–2017 to reflect the environment which workers are exposed to. Secondly, outdoor workers (70 from Greece, 216 from Slovenia) provided a self-assessment of heat stress. The heat wave timeline is presented as an effective way of illustrating long-term changes in heat waves’ characteristics for various stakeholders. In both countries, workers assessed as significant the heat stress impact on productivity (Greece 69%, Slovenia 71%; *p* > 0.05), and in Slovenia also on well-being (74%; *p* < 0.01). The main experienced symptoms and diseases were thirst (Greece 70%, Slovenia 82%; *p* = 0.03), excessive sweating (67%, 85%; *p* = 0.01), exhaustion (51%, 62%; *p* > 0.05) and headache (44%, 53%; *p* > 0.05). The most common way to reduce heat stress was drinking more water (Greece 64%, Slovenia 82%; *p* = 0.001). Among the informed workers, the prevalent source of information was discussions. Therefore, educational campaigns are recommended, together with the testing of the efficiency of mitigation measures that will be proposed on the Heat-Shield project portal.

## 1. Introduction

Heat waves are typically known as prolonged periods of excessive heat or “summertime episodes with extremely high surface air temperatures and lasting for several days or longer” [1]. Several studies have assessed that heat waves are becoming more intense, more frequent, and lasting longer [2,3,4,5]; and across the globe, they have been linked with substantial increase in morbidity and mortality [6].

The Intergovernmental Panel on Climate Change [7] classified heat waves as one of the most extreme weather events associated with climate change. Studies of heat waves’ climatology provide important background for the research of several heat wave consequences, such as increasing heat stress among the general and working population, because their knowledge, behavior, and acclimatization, in general, reflect the environment they have been living in. Thus, analyses of the long-term occurrence of heat waves at specific locations, including changes due to climate change, help better understand the variability in the assessment of heat stress, the perceived symptoms, and workers’ knowledge.

The number of vulnerable people exposed to heat waves around the world increased by about 125 million between 2000 and 2015. In the same period, the productivity of the outdoor working population decreased by 5.3% worldwide [8]. Hot working environments are considered to be an occupational health problem affecting the body’s major organs, including heart, kidneys, and brain [9]. However, even before the manifestation of clinical symptoms, heat exhaustion has a negative impact on workers’ ability to carry out physical and mental work [10], especially continuous work on an hourly basis. Consequently, apart from the detrimental effects of heat waves on human health, reduced labor productivity and economic loss are also among the serious outcomes. When productivity becomes affected, company management may show a particularly high incentive to address the heat issue [11], but the development and evaluation of preventive measures depend also on the quality of heat stress assessments [12].

The successful design and implementation of a prevention plan require an effective and complete heat warning system based on critical thresholds [13]. According to a comprehensive assessment of the development of heat preparedness planning among the 53 WHO European Region member states, using a unique methodology based on criteria developed and pre-tested by the WHO, only 18 countries have developed heat-health action plans [3]. However, while there are some improvements and actions in the eyes of the general public, during the last decade, only 7 out of 12 European countries that had prepared heat wave early warning systems described outdoor workers as a risk group [14]. The recent systematic review and meta-analysis by Flouris et al. [15] showed that occupational heat stress has serious health and productivity outcomes and should be recognized as a public health problem. Europe cannot afford to overlook workers, who are vulnerable especially in economic sectors like agriculture [16,17,18], construction [19], and the manufacturing industry [20].

In this light, the purpose of the study was, first, to analyze the climatological background of heat waves and outdoor working conditions in two climate-heterogeneous European countries, Slovenia and Greece. Additionally, we obtained Slovenian and Greek outdoor workers’ self-assessments of their heat stress symptoms, heat-induced diseases, and their own capacity to reduce their exposure to heat stress. Our aim was to compare both countries to identify the main differences and similarities between them with respect to the occurrence of heat waves, their impacts, and workers’ perceptions of heat stress.

## 2. Materials and Methods

### 2.1. Heat Wave Climatology

The impact and the perception of a heat wave vary considerably from region to region. For example, multi-day air temperatures above 30 °C in summer in Central or even North Europe would be expected to create more problems than similar temperatures in South Europe [21]. For this reason, heat waves should be defined based on a high percentile base and not on absolute air temperature thresholds, since the latter method does not ensure that local climatology and acclimatization have been assessed. In our case, two European countries, Slovenia and Greece, with distinct differences in climate and weather conditions, were included in the study. To perform a detailed analysis of the heat wave events in the two countries, we examined data from two meteorological stations, Brnik (Airport Ljubljana, Slovenia; 46°13′4″ N, 14°28′39″ E, 364 m a.s.l.) and Larisa (Greece; 39°38′19″ N, 22°24′32″ E, 85 m a.s.l.). For Slovenia, the weather data were obtained from the Slovenian Environment Agency’s archive, and for Greece from the ECA&D dataset [22].

Lacking a universal definition of heat waves, national meteorological offices have adopted their own definitions in order to be able to issue heat alarms. In Slovenia, a new definition of a heat wave was developed by the Slovenian Meteorological Society, the Slovenian Environment Agency, the National Health Institute, and the Biotechnical Faculty, based on the daily mean air temperature (*Tmean*): In the continental part of Slovenia, a heat wave occurs when *Tmean* is equal to or exceeds 24 °C for at least three days [23]. The threshold approximately coincides with the 97th percentile of *Tmean* in the period 1986–2015 and with the 96th percentile of *Tmean* in the period 2006–2015; thus, the same percentile was used to set the threshold of 28.5 °C for Larisa (Greece).

Since the scientific definitions and the graphical presentations of heat waves are often too complicated for the general public and workers to understand, an effective way of illustrating all the main characteristics (quantity, intensity, duration, date of occurrence) of heat waves is proposed. In addition, a comparison of annual temperature sums (*Tsum*) has been performed. *Tsum* is the cumulative *Tmean* excess above the threshold during all heat waves in one year. According to Kysely [24], temperature sum is probably the most appropriate parameter to determine the severity of a heat wave.

### 2.2. Workers’ Perception of Heat Stress

In the context of the HEAT-SHIELD project [25], a cross-sectional study has been carried out in both countries, as mentioned above. A slightly modified questionnaire, previously utilized in the HOTHAPS (The High Occupational Temperature, Health, and Productivity Suppression) Program [26], was used, aiming specifically at workers’ perception of heat stress. The survey was conducted in Slovenia in 2017, and in Greece in 2018. A sample of more than 800 workers from various industrial sectors (mainly agriculture, tourism, and construction) was asked to participate in the study, providing verbal consent and answering in their own language. This analysis considered all those who spent more than one-third of their daily working time outdoors (286), 216 from Slovenia and 70 from Greece. The questionnaire included questions on the demographics, the workers’ knowledge of the companies’ heat plans, perceived symptoms of heat stress or heat-induced illnesses and possible protective measures. Respondents were provided with possible answers.

Results were compared between the two countries using Pearson Chi-square tests and Independent Samples t-tests. A *p*-value (*p*) lower than 0.05 was considered to be statistically significant. Data were analyzed using the software SPSS for Windows.

## 3. Results

### 3.1. Comparison of Heat Waves in Slovenia and Greece

**Figure 1:**
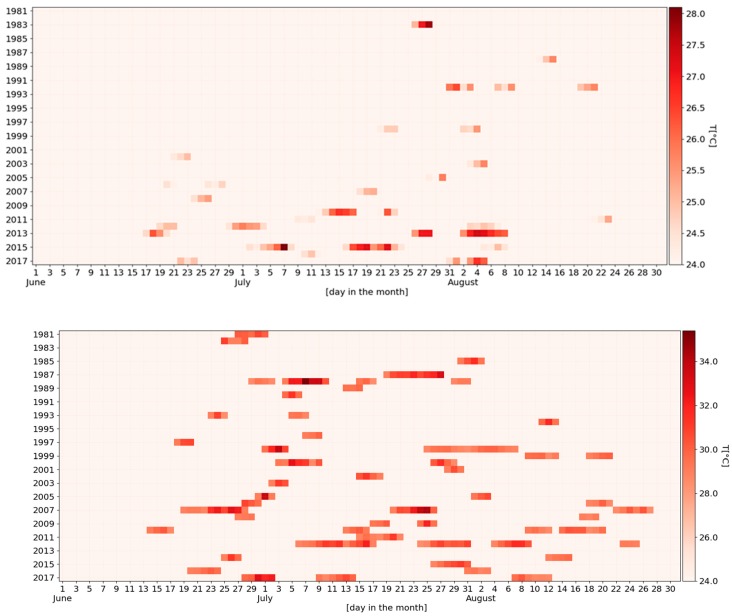
A daily overview of heat waves and their intensity in the period 1981–2017 in Brnik (Slovenia; top) and Larisa (Greece; bottom) from 1 June to 31 August. Only days in heat waves are colored, darker red designating higher daily mean air temperatures.

As mentioned above, the percentile-based thresholds for the occurrence of a heat wave were 24 °C for the central part of Slovenia and 28.5 °C for Larisa, Greece. Despite the fact that the threshold was much higher in Greece, a greater number of heat waves was observed throughout the examined period (Figure 1). During the heat waves, the average daily air temperature reached a maximum of 28 °C (4 °C above the threshold) in Slovenia and 35 °C (6.5 °C above the threshold) in Greece. The number, duration and distribution of heat waves throughout the summer season have been more similar in the two countries in the recent years than previously.

**Figure 2:**
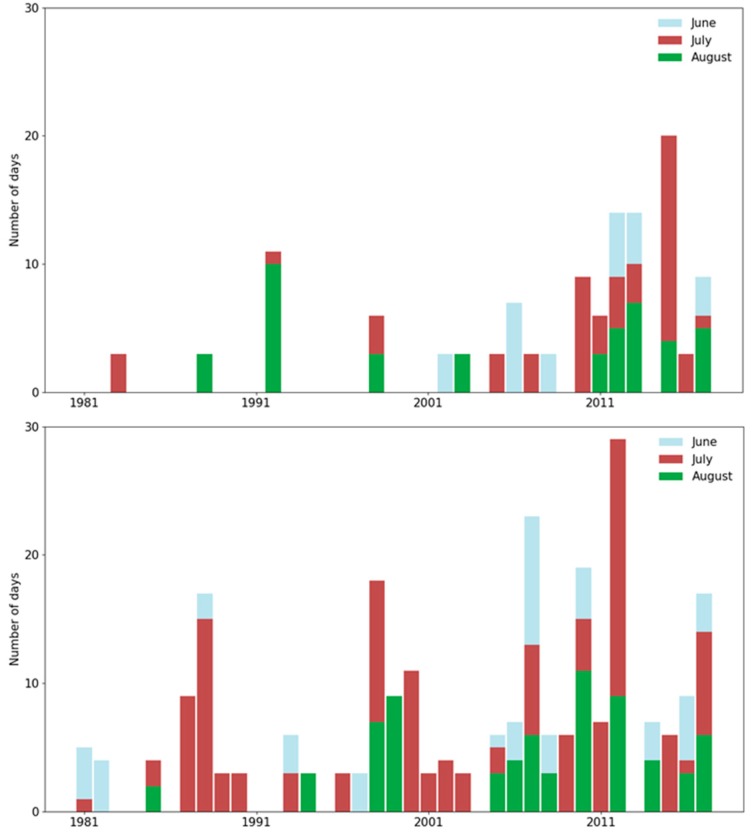
The annual number (divided monthly by colors) of days in heat waves in the period 1981–2017 in Brnik (Slovenia; top) and Larisa (Greece; bottom).

**Figure 3:**
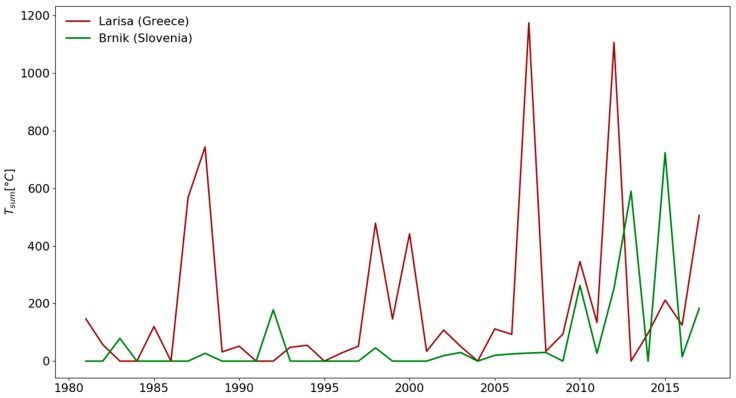
The heat waves’ temperature sums (above the threshold) for the period 1981–2017 in Brnik (Slovenia) and Larisa (Greece).

From 1981 to 2000, heat waves were common in the summer months in Larisa, while in Brnik they were restricted to few extreme days in July and August. In most of the examined period, the duration of heat waves was much longer in Larisa than in Brnik (Figure 2). Moreover, the longest duration of heat waves was observed in the month of July in both countries. If the annual heat waves’ temperature sum (Figure 3) in Brnik was very low (almost zero) for a long period of time (1981–2009), it has dramatically increased in recent years (2010, 2013, 2015, and 2017). On the other hand, in the city of Larisa, much higher temperature sums were sporadically distributed throughout the period 1981–2017.

### 3.2. Worker’s Self-Assessment of Heat Stress Impacts

A total of 286 workers were assessed, 70 from Greece and 216 from Slovenia. In both countries, the highest percentage of workers participating in the study were male, the highest share of workers were in the age group of 31 to 40 years, and the education level of workers in both countries was similar. As it has been mentioned in the methodology, only workers who spend at least one-third of their working hours outdoors were analyzed. In both countries, approximately half of them worked outdoors one to two-thirds of their working time while the other half worked outdoors two to three-thirds of their time. The majority of workers in both countries were healthy, while Slovenian workers had a higher rate of chronic disease in comparison to Greek workers. The characteristics of the sample are presented in Table 1.

**Figure 4:**
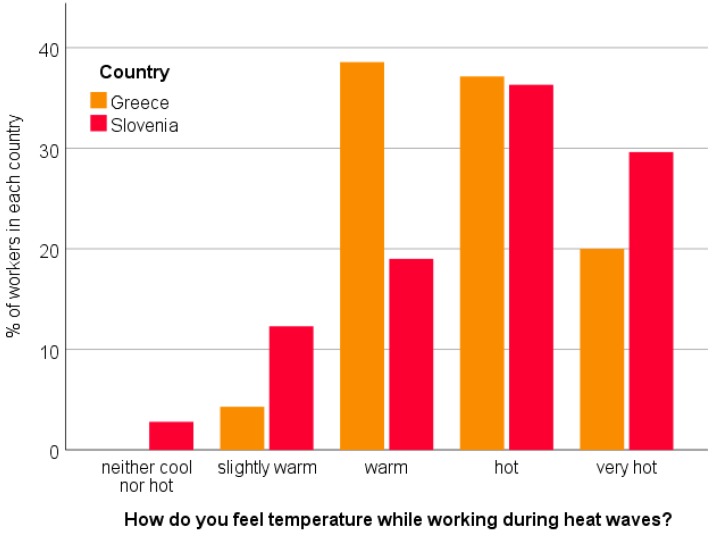
Workers’ assessment of their workplace temperature conditions during heat waves.

Regarding the workers’ assessment of workplace temperature conditions during heat waves, the vast majority of Greek (~95%) and Slovenian (~85%) workers described their workplace conditions as warm, hot and very hot. (Figure 4). However, in Slovenia, a small percentage of the workers (~15%) characterized the working conditions as “neither cool nor hot” or “slightly warm”.

In both countries, the vast majority of the workers said that the heat stress impact was not negligible for their work (Greece 93%, Slovenia 94%; *p* > 0.05) (Table 2). Furthermore, a high share of the workers (Greece 69%, Slovenia 71%; *p* > 0.05) assessed the heat stress impact on productivity as significant. According to the workers’ report, heat stress also had a significant impact on their well-being (a statistically significant difference between the percentages of workers in Greece (60%) and Slovenia (74%); *p* = 0.03), but not on their concentration (Greece 74%, Slovenia 62%; *p* > 0.05). All the stated impacts are statistically significant (*p* < 0.01) for the whole sample and for each country separately, with the impact on well-being in Greece being the only exception.

Moreover, in both countries, the main experienced symptoms of heat stress were thirst (Greece 70%, Slovenia 82%; *p* = 0.03) and excessive sweating (Greece 67%, Slovenia 85%; *p* = 0.01), followed by tiredness (Greece 51%, Slovenia 62%; *p* > 0.05). Only 3% of Greek workers did not perceive any symptoms during heat waves and none in Slovenia. A similar pattern (not significant) can be noticed with experienced heat induced diseases, where the most common ones were exhaustion (Greece 51%, Slovenia 62%; *p* > 0.05) and headache (Greece 44%, Slovenia 53%; *p* > 0.05). However, in Greece there were more instances (difference statistically significant) of heat cramps (4%, 0.5%; *p* = 0.02) and also more examples of heat stroke (6%, 0%; *p* < 0.001). Even though these two are the most serious problems, there were slightly fewer hospitalizations due to heat stress in Greece (6%, in Slovenia 7%; *p* > 0.05). All reported occurrences of heat stress symptoms, heat-induced diseases, and hospitalizations are statistically significant (*p* < 0.01) for the whole sample and both countries separately, with the exception of tiredness and exhaustion in Greece, and headache in all three samples.

On the other hand, the share of workers who do not see any options to reduce their own exposure to heat stress is significantly higher among Greek workers than Slovenian (24% and 13%; *p* = 0.02). In both countries, drinking more water was found as the most common way to reduce heat stress (64%, Slovenia 82%; *p* = 0.001). This was followed by measures including adjustment of the working schedule (Greece only 34%, Slovenia 66%; *p* < 0.001), retreat to a cooler place during breaks (Greece 41%, Slovenia 54%; *p* > 0.05), wearing more appropriate clothes (Greece 40%, Slovenia 49%; *p* > 0.05) and increasing the number of breaks during the workday, which is also much less common in Greece (Slovenia 33%, Greece 20%; *p* = 0.04). The shares of workers using the measures ‘retreat to a cooler place’ and ‘wearing more appropriate clothes’ (*p* > 0.05) are statistically significant neither for Greece nor for Slovenia, or for the whole sample, while the other shares are (*p* < 0.02).

**Figure 5:**
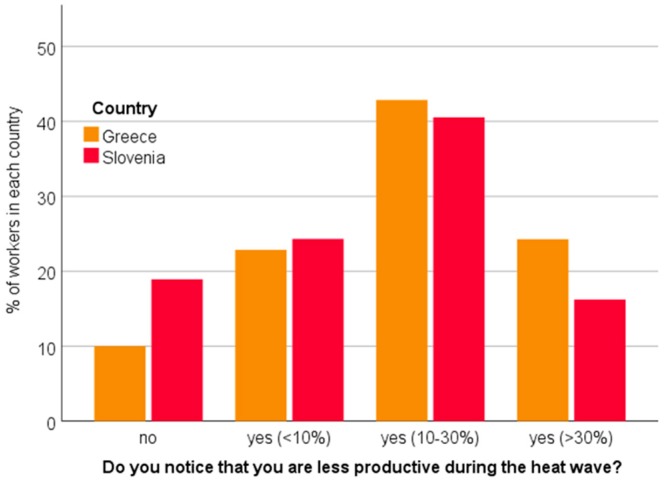
Workers’ assessment of their reduced productivity due to heat stress during heat waves.

Very similar percentages of workers from both countries reported either a small impact on productivity—reduced by less than 10% (more than 20% of workers from each country); or a medium impact—10 to 30% fall in productivity (more than 40%; Figure 5).

**Figure 6:**
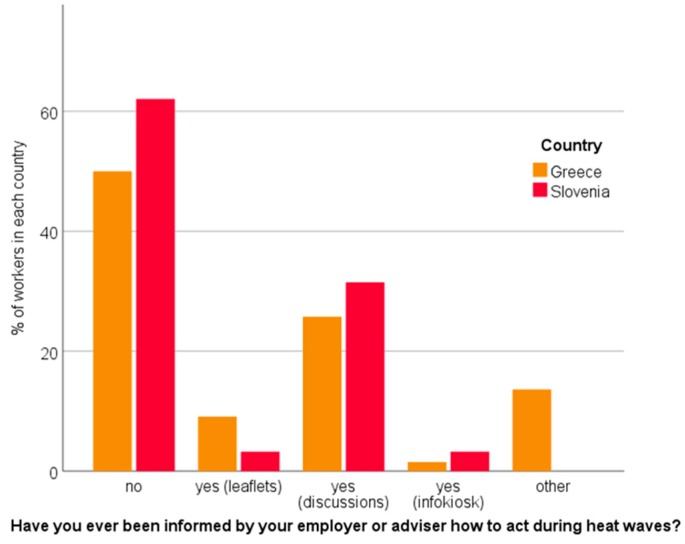
Workers’ reports on how they were (or were not) informed about heat stress.

On the other hand, more Greek workers (24%, in Slovenia 16%; *p* > 0.05) assessed the loss of productivity to be higher than 30%. The study also revealed that the majority of both Greek (~50%) and Slovenian workers (~60%) were not informed by their employer about how to act during heat waves. Among the informed workers, ‘discussions’ seem to be the most prevalent source of information in both countries (Greece: ~25%, Slovenia: ~30%) (Figure 6).

## 4. Discussion

The observed changes in the number, duration and intensity of heat waves (e.g., [4,20]), which coincide with the perceived effect on the health and well-being of workers [15], mark an important guideline for European research. The climatological background of heat waves in two regions in Slovenia (Brnik) and Greece (Larisa) was analyzed and used as a framework to understand different outdoor working conditions and acclimatization or knowledge bases. Questionnaire-based research among outdoor workers provided an overview of the already experienced problems and the ways of reducing heat stress.

The first part of the study showed the main differences in the climates of Brnik and Larisa in terms of heat waves. Daily mean air temperatures were much higher in Larisa and heat waves were already present in the earlier years of the period 1981–2017. Although the definition of a heat wave is still an unresolved issue within the scientific community (e.g., [3,4,8,24,27]), the analysis followed the recommendation of Pascal et al. [13] to use a high percentile of the meteorological indicators’ distribution. The comparison of the heat waves’ characteristics in Brnik and Larisa looks similar to the report of Kuglitsch et al. [28], who found that in the eastern Mediterranean, the intensity, length, and number of heat waves have increased by a factor of 6 to 8 since the 1960s. Heat waves that are unusual in the current climate will become more common along with rising global mean temperatures, and could occur in any country in Europe [4]. To prepare prevention plans and share this information, an appropriate means of public communication is needed, as the general public may not understand the technical meteorological jargon. In Slovenia, the heat wave timeline (Figure 1) seems to work fine for this purpose, as it combines a lot of information about each heat wave and is easy to understand. Therefore, we recommend its use when explaining the climatology of heat waves to employers and workers, which is a necessary step in expanding their knowledge of the topic and helping them understand that the problem is escalating and how important it is to start implementing mitigation and adaptation measures.

The second part provides an important insight into how wide the common range of experienced health problems already is, and shows that productivity loss due to heat stress was noted. Working people are particularly affected by high heat exposures in occupational settings [15], and this situation will become harder as climate change progresses [29]. As older adults have a higher susceptibility to heat stress [30], not only the increase in temperature but also the aging of the population may contribute to higher numbers of people sensitive to heat—however, the acclimatization of the population is also possible [13]. On the other hand, older workers are more experienced and more familiar with the work, and hence more efficient [19]. According to Luther et al. [31], risk factors for heat-related illnesses are mainly strenuous outdoor physical activity during the hottest day-time hours, age above 64 years, limited access to air-conditioning, medication and substance use (alcohol), mental illness, physical disability/impaired mobility, cognitive impairment, heart and lung disease. Significant positive associations between temperature and acute work-related injury are also known for a wider range of vulnerable worker subgroups, with the odds of injury increasing by 0.8% for each 1 °C increase in daily maximum temperature [32].

Although the climates of both cities differ in heat intensity, the results showed that workers from both countries assessed heat stress impact on productivity as significant. Additionally, a slightly higher percentage of Greek workers (not statistically significant) assessed the loss of productivity due to heat stress to be higher than 30%. The majority of respondents considered heat stress to have no impact on concentration, but to significantly affect well-being. Symptoms and diseases are mainly the same as described by Luther et al. [31] to be the strongest indicators of heat stress: fainting, nausea and/or vomiting, confusion, dizziness, diarrhea, headache, irritability, hallucination, loss of coordination, weakness. There are several factors such as humidity, demography or people’s adaptation to heat that may explain why a similar anomaly of temperature results in different health outcomes [13]. It is interesting that despite lower temperatures, fewer and shorter heat waves, a statistically significant higher share of Slovenian workers perceived the impact on well-being, and experienced thirst, excessive sweating, and tiredness, followed by more heat-induced diseases than in Greece. The only exceptions are heavier disease occurrence, like muscle and heat cramps and heat stroke, which prevailed in Greece, the reason possibly being the higher temperature extremes. There were slightly more hospitalizations due to heat stress in Slovenia, which could be due to the fact that Greek workers are more accustomed to the hot climate, as it has not changed as much as in Slovenia in the last decade or two.

Considering the stated high impacts on the productivity and well-being of workers, it is urgent to provide measures or guidelines to mitigate the impacts of heat stress. First of all, there is still room for improvement in the provision of information, as more than half of the workers have not yet been informed about possible impacts and actions during heat waves. At a later stage, even when the knowledge level of individuals is good, there can be a misconception of people’s own risk [33] or abilities. Even if ‘drinking more water’ was most commonly and naturally used as the measure to lower heat stress, it does not mean that workers did, in fact, drink enough water. The study by Piil et al. [34] showed that ~70% of workers in Europe often come to work dehydrated and stay dehydrated until the end of the work-shift. This is in line with a recent meta-analysis assessing global literature, which reported that urine specific gravity is increased by 14.5% during a single work shift under heat stress [15]. It is not surprising, therefore, that 15% of individuals working in heat stress conditions suffer from kidney disease or acute kidney injury [15]. This is markedly higher compared to the prevalence rates reported for kidney disease (10%) [35], as well as for acute kidney injury in high- (2%) [36,37] and low- (3–9%) [38,39] income countries. Taken together, these results raise serious concerns for the kidney function of individuals typically/frequently working in heat stress conditions, because even a single episode of acute kidney injury can lead to chronic kidney disease with significant socioeconomic and public health outcomes [40].

Despite our best intentions, it is important to note that the results obtained in this study should be considered through the prism of certain limitations. For instance, the results are not necessarily representative for Greece and Slovenia as only two stations were included in the analysis of heat waves and due to the small size of the samples. However, the research gives a first indication of the heat stress perception in these countries. Also, the workers’ self-assessments may be too subjective or misinterpreted, but the majority is consistent with the already detected impacts of heat stress.

## 5. Conclusions

Our study showed the significance of heat stress impacts on outdoor workers, bearing in mind that the data is limited to two stations in Greece and Slovenia. The assessed high negative impact on productivity and well-being, endangering health, is consistent with the other findings of the Heat-Shield project, where we aim to improve both health and productivity. An exchange of experiences and lessons learned can substantially accelerate and improve the process of ensuring more heat resilient working conditions in Europe. In communication with health institutions, policymakers, employers, workers or others outside of the scientific field, it is of great importance to find out how to communicate results in a clear and easy to understand way. Here, the representation of heat waves used in this paper can be a good example, illustrating the increase in the frequency, intensity, duration and time span of heat waves to the point.

The comparison of two stations in Greece and Slovenia showed higher temperatures and more heat waves in Greece, but more noticeable changes in this field in Slovenia. Therefore, the lower degree of acclimatization among Slovenian outdoor workers may be one of the main reasons they have more often described conditions as very hot and perceived more heat stress symptoms and heat-induced diseases than Greek workers. On the other hand, much higher temperature extremes in Greece could be the reason higher productivity loss was perceived in Greece and why some heavier diseases were experienced mainly there. As workers in both locations share a similar level of knowledge about heat stress, educational campaigns are needed and further research of the problem, which includes the testing of the efficiency of several mitigation measures, will be proposed on the Heat-Shield project portal.

## Figures and Tables

**Table 1:** A comparison of the questionnaire’s results in Greece and Slovenia—description of the samples.

	Greece (*n* = 70)	Slovenia (*n* = 216)
**Gender**		
Male	77.1%	57.4%
Female	22.9%	42.6%
**Age**		
20–30	20.0%	14.8%
31–40	44.3%	30.6%
41–50	10.0%	23.1%
51–60	15.7%	22.2%
61 and more	10.0%	9.3%
**Education level**		
Primary school or none	20.0%	5.6%
Secondary school	21.4%	43.5%
College	25.7%	26.4%
Bachelor’s degree	24.3%	21.3%
Master’s degree/PhD	8.6%	3.2%
**Working outdoors**		
one–two thirds	44.3%	50.9%
two–three thirds	55.7%	49.1%
**Chronic disease**		
Yes	15.7%	30.6%
No	84.3%	69.4%

**Table 2:** A comparison of the questionnaire’s results in Greece and Slovenia—personal experiences of workers (* statistically significant difference between the countries).

	Greece (*n* = 70)	Slovenia (*n* = 216)	*p*-Value
**Heat stress impact is negligible**			
Yes	7.1%	6.0%	0.763
No	92.9%	94.0%	
**Heat stress has a significant impact on well-being**			
Yes	60.0%	74.1%	0.025 *
No	40.0%	25.9%	
**Heat stress has a significant impact on concentration**			
Yes	25.7%	38.0%	0.062
No	74.3%	62.0%	
**Heat stress has a significant impact on productivity**			
Yes	68.6%	71.3%	0.664
No	31.4%	28.7%	
**Experienced heat stress symptoms**			
None	2.9%	0.0%	0.013 *
Thirst	70.0%	82.4%	0.026 *
Tiredness	51.4%	62.0%	0.116
Dizziness	18.6%	24.1%	0.340
Confusion	15.7%	10.6%	0.255
Enhanced stress	12.9%	25.9%	0.023 *
Excessive sweating	67.1%	84.7%	0.010 *
**Experienced heat induced diseases**			
Headache	44.3%	52.8%	0.217
Exhaustion	51.4%	62.0%	0.116
Prickly heat	5.7%	13.0%	0.095
Muscle cramps	7.1%	6.0%	0.736
Fainting	4.3%	8.3%	0.259
Nausea/vomiting	5.7%	13.9%	0.066
Heat cramps	4.3%	0.5%	0.018 *
Heat stroke	5.7%	0.0%	<0.001 *
None	22.9%	15.3%	0.144
**Hospitalization due to heat stress**			
No	94.3%	92.6%	0.629
Yes	5.7%	7.4%	
**Can you reduce your exposure to heat stress**			
No	24.3%	12.5%	0.018 *
Yes	75.7%	87.5%	
**How can you reduce your exposure to heat stress**			
Increase the number of breaks during the work	20.0%	32.9%	0.041 *
Retreat to cooler space during breaks	41.4%	54.2%	0.064
Wear appropriate clothes	40.0%	49.1%	0.186
Drink more water	64.3%	82.4%	0.001 *
Adjust working schedule	34.3%	65.7%	<0.001 *
